# Manufacturing and Examination of Vaginal Drug Delivery System by FDM 3D Printing

**DOI:** 10.3390/pharmaceutics13101714

**Published:** 2021-10-16

**Authors:** Petra Arany, Ildikó Papp, Marianna Zichar, Géza Regdon, Mónika Béres, Melinda Szalóki, Renátó Kovács, Pálma Fehér, Zoltán Ujhelyi, Miklós Vecsernyés, Ildikó Bácskay

**Affiliations:** 1 Department of Pharmaceutical Technology, Faculty of Pharmacy, University of Debrecen, Nagyerdei Körút 98, H-4032 Debrecen, Hungary; arany.petra@euipar.unideb.hu (P.A.); feher.palma@pharm.unideb.hu (P.F.); ujhelyi.zoltan@pharm.unideb.hu (Z.U.); vecsernyes.miklos@pharm.unideb.hu (M.V.); 2 Doctoral School of Pharmaceutical Sciences, University of Debrecen, Nagyerdei Körút 98, H-4032 Debrecen, Hungary; 3 Department of Computer Graphics and Image Processing, Faculty of Informatics, University of Debrecen, Kassai út 26, H-4028 Debrecen, Hungary; papp.ildiko@inf.unideb.hu (I.P.); zichar.marianna@inf.unideb.hu (M.Z.); 4 Institute of Pharmaceutical Technology and Regulatory Affairs, University of Szeged, Eötvös u. 6, H-6720 Szeged, Hungary; geza.regdon@pharm.u-szeged.hu; 5 Department of Medical Imaging, Faculty of Medicine, University of Debrecen, Nagyerdei Krt. 98, H-4032 Debrecen, Hungary; beres.monika@med.unideb.hu; 6 Department of Biomaterials and Prosthetic Dentistry, Faculty of Dentistry, University of Debrecen, Nagyerdei Körút 98, H-4032 Debrecen, Hungary; szaloki.melinda@dental.unideb.hu; 7 Department of Medical Microbiology, Faculty of Medicine and Pharmacy, University of Debrecen, Nagyerdei Körút 98, H-4032 Debrecen, Hungary; kovacs.renato@med.unideb.hu

**Keywords:** personalized medication, vaginal ring, 3D printing, FDM, dissolution test, biocompatibility, MTT assay

## Abstract

Vaginal drug delivery systems can provide a long-term and constant liberation of the active pharmaceutical ingredient even for months. For our experiment, FDM 3D printing was used to manufacture the vaginal ring samples from thermoplastic polyurethane filament, which enables fast manufacturing of complex, personalized medications. 3D printing can be an excellent alternative instead of industrial manufacturing, which is complicated and time-consuming. In our work, the 3D printed vaginal rings were filled manually with jellified metronidazole or chloramphenicol for the treatment of bacterial vaginosis. The need for manual filling was certified by the thermogravimetric and heatflow assay results. The manufactured samples were analyzed by an Erweka USP type II Dissolution Apparatus, and the dissolution profile can be distinguished based on the applied jellifying agents and the API’s. All samples were considered non-similar based on the pairwise comparison. The biocompatibility properties were determined by prolonged MTT assay on HeLa cells, and the polymer could be considered non-toxic. Based on the microbiological assay on *E. coli* metronidazole and chitosan containing samples had bactericidal effects while just metronidazole or just chitosan containing samples bacteriostatic effect. None of these samples showed a fungistatic or fungicide effect against *C. albicans.* Based on our results, we successfully manufactured 3D printed vaginal rings filled with jellified metronidazole.

## 1. Introduction

Since the first patent of the vaginal ring as a possible drug delivery system in 1970 [1], different vaginal rings had been approved [2], which are mostly used as contraceptives to provide a long-acting effect, including the opportunity to initiate or discontinue use whenever desired [3]. As these drug delivery systems do not provide protection against sexually transmitted diseases, a new approach has been considered, and as a result, dapivirine containing vaginal rings were approved by EMA in 2020, which provides risk reduction of HIV-1 infection [4].

The design and manufacturing methods vary according to each product because neither guidelines nor regulations are provided by the medical agencies. The drug delivery systems can be matrix or reservoir types manufactured by injection molding, hot-melt extrusion, or additive manufacturing as well [5]. 

Additive manufacturing or 3D printing encompasses a wide range of processes and is used for the manufacturing of a wide variety of drug delivery systems [6]. Fused deposition modeling (FDM) technology is the most commonly used technology and enables the manufacturing of oral dosage forms [7,8], intrauterine systems [9], implants [10,11] or vaginal rings [5,12]. 

Vaginal infections are extremely widespread. Even though these infections are not resulting in high mortality, but can be long-lasting and difficult to cure, which can result in high levels of anxiety and decrease the quality of life [13]. Relapsing is a serious problem due to the particular anatomy of the vagina and to the bacterial diffusion between the rectum and the vagina, thus, the intestinal tract becomes a pathogen microorganism reservoir and plays an important role in the infection reappearance [14].

Bacterial vaginal infections can be treated both orally or locally. For the oral treatment, mostly clindamycin and metronidazole are used. Despite the majority of women being effectively treated with these antimicrobials, 30% will experience a recurrence after 4 weeks of treatment due to the incomplete eradication of pathogens, unsuccessful reestablishment of lactobacilli flora, or resistance development [13]. The local administration of different vaginal suppositories dates back to the 1980s in Hungary, and the gynecologists mostly prescribe magistral formula of chloramphenicol, nystatin, sulfadimidine or metronidazole combinations (named as suppository CNS or CNS + M) [15].

Chloramphenicol (CAP) is a broad-spectrum and widely-used antibiotic that has a good inhibitory effect on brucella, Gram-positive bacteria, Gram-negative bacteria, rickettsia, and chlamydia. The resistance development is small-scale compared to other antibiotic agents [16,17]. The API belongs to class III according to the Biopharmaceutical Classification System (BCS) [18]. The most important concerns that limit the utility of this antibiotic are the adverse effects, such as neurotoxicity, bone marrow depression and in some cases, severe aplastic anemia [19]. The chloramphenicol can be easily characterized by UV-VIS spectroscopy at the wavelength of 273.8 mm [20].

Metronidazole is the first-line therapy against bacterial vaginosis and is used both orally and locally [21]. The API is a model class I drug according to the BCS System [22]. Side effects of metronidazole include metallic taste, nausea (in 10% of patients), transient neutropenia (7.5%), disulfiram-like effect with alcohol, prolongation of international normalized ratio, and peripheral neuropathy, but these unwanted effects can be eliminated with local administration [23]. The metronidazole’s UV-VIS spectra can be characterized at 320 nm wavelength, but at lower pH the maximum can be at 280 nm [24].

For the manufacturing of a drug delivery system with 3D printing, researchers incorporate the API into the filament with hot-melt extrusion, and then the API-containing filament is printed with FDM technology [25]. The high extrusion temperature used in FDM (more than 120 °C) and in hot-melt extrusion (more than 150 °C) enables only heat-stable APIs incorporation into the polymeric filament [26]. Polymer modification [27] or different added excipients can also decrease the printing temperature, but the lowest used printing temperature was 165 °C, which is still not adequate for a lot of API’s [28]. Even though many research projects are trying to solve the problem of high printing temperature, the most promising solution would be to manufacture a carrier system in which all types of APIs can be incorporated [29].

Our innovative idea was to manufacture the vaginal rings as carrier systems by 3D printing and then fill them with jellified API’s. Vaginal gels are easy to manufacture, comfortable, and have the ability to cover the surface of mucosal membrane and to display the effect on the vaginal mucosa. The use of mucoadhesive polymers can improve the contact time with the mucosa, delay the loss of the formulation, enhance the bioavailability, prolong the effect, cause fewer side-effects and increase patient compliance [30,31].

Drug release of vaginal formulations can differ on the test method, the test medium type and amount, the agitation, and the temperature. Most research groups use 60 rpm, 37 °C, and vaginal fluid simulant (recipe by Owen and Katz). Since none of the major international pharmacopeias mentions dissolution/drug testing for vaginal rings, thus compendial dissolution methods do not exist [32].

The biocompatibility properties of the printed samples were measured by a prolonged MTT cytotoxicity test. MTT assay is a broadly used, rapid colorimetric method to measure the in vitro cytotoxicity of certain compounds, and in this case, even the dissolved xenobiotic can be detected [33]. Several assays may be used, such as MTT assay, LDH test, real-time cell electronic sensing assay (RT-CES), etc., but the ISO standard describes all parameters adequately for the MTT test [34].

The evaluation of antimicrobial effectiveness using reference bacteria strains, fungi strains, or strains obtained from clinical samples has been performed for decades [35]. In the case of metronidazole the antimicrobial efficacy was tested against *E. coli* because this pathogen can cause bacterial vaginosis as a Gram-negative, anaerobic bacteria [21].

Our aim was to manufacture a vaginal ring as a carrier system by FDM 3D printing and then easily fill these pre-printed samples with the jellified API’s (vaginal gels) depending on the patient’s need. This article focuses on avoiding the loss and decomposition of API through the printing process and manufacturing a drug delivery system, which can be directly printed at the bedside or in the pharmacy. Thus, the preparation is more easily and rapidly accessible for the patients need, which result in increased patient adherence.

## 2. Materials and Methods

### 2.1. Materials

#### 2.1.1. Used Polymer Filaments

For the 3D printing process, polylactic acid (PLA), PLA Gypsum, and PLA Foam were purchased from Philament Kft. (Miskolc, Hungary). Thermoplastic polyurethane (TPU) 80A LF was purchased by Ultrafuse FFF (Emmen, The Netherlands). The filament diameters were 1.75 mm. The properties of the commercially available PLA and TPU filaments are compared in Table 1, and the properties of the 3D-printed filaments are provided in Table 2 below. The data were provided by Philament Kft and Ultrafuse FFF.

#### 2.1.2. Jellifying Agents

Carbopol 934 was a gift from Lubrizol GmbH (Ritterhude, Germany). Medium molecular weight chitosan (CAS:9012-76-4) was purchased from Sigma Aldrich Co. (Merck Kft., Budapest, Hungary). Hydroxyl ethyl cellulose (CARN:9004-62-0) was purchased from Molar Chemicals Kft (Halásztelek, Hungary). Agar-agar was purchased from Discovery Bliss Kft. (Csömör, Hungary).

#### 2.1.3. Model API’s

Chloramphenicol and metronidazole were purchased from Molar Chemicals Kft. (Halásztelek, Hungary).

### 2.2. Methods

#### 2.2.1. Design of the Drug Reservoirs and Printing of the Samples

The digital models of the samples were designed using SolidWorks (Dessault Systèmes, Vélizy-Villacoublay, France), which is a modeling computer-aided design and engineering software. Exporting the digital design into a stl (standard tessellation language) file makes it directly printable on the 3D printer [29].

The shape was based on the commercially available vaginal ring—NuvaRing^®^, but the edges were chamfered because it was more beneficial for the 3D printing process. Each sample was 5 mm tall and printed in two different diameters (39.19 and 41.19 mm) to provide the perfect fitting. In our article, we referred to these two diameters as lower and upper parts. All the other geometric and additional properties of the samples were determined in the slicing software [36]. Printing parameter adjustment was a critical process, which will affect the mechanical properties of the samples [37]. In our research, the used extrusion width was 0.400 mm, the layer height 0.100 mm, vertical shell 5 loops, top/bottom shell concentric, 10/10 shell.

Batches of 4 drug delivery systems were printed on a Craftbot 3 3D Printer (CraftUnique Kft., Budapest. Hungary). The parameters of the 3D printing process are summarized below (Table 3).

#### 2.2.2. Gel Formation and Sample Manufacturing

Four different formulations were prepared. The first formulation was prepared from Carbopol 934 and a 1 *w/w*% solution was manufactured with distilled water at 500 rpm shaking [21]. The second and third formulation was prepared by medium molecular weight chitosan and hydroxyethyl cellulose (HEC). The second formulation consisted of 3 *w/w*% chitosan and 4 *w/w*% HEC and the third formulation of just 1 *w/w*% chitosan and 4 *w/w*% HEC with distilled water, 300 rpm shaking and manual mixing [14,30]. The last formulation was designed by our research group and a 1 *w/w*% agar-agar solution was prepared with distilled water and heating up to 100 °C.

The sample manufacturing was based on that the vaginal ring was pre-printed in the 3D printer to manufacture a lower and upper part separately. Then 5 g of chloramphenicol or metronidazole was measured on an analytical balance MettlerToledo AX105 DeltaRange (Colombus, OH, USA), and it was suspended in a mortal with 20 g of gel. Then, the suspensions were put into a syringe. The lower part of the vaginal ring was tared on the analytical balance, and 1.5 g gel was filled by the syringe to every lower part. Finally, the upper part was put and closed manually.

#### 2.2.3. Weight Variation and Content Uniformity

Before the experiments, 10 samples from each formulation were individually weighed, and the calculated average with standard deviation gave the weight variation. Then the samples were opened for content uniformity determination. The samples were separately immersed in 200 mL of simulated vaginal fluid and stirred for 60 min. The drug amount was determined using UV spectrophotometry with ThemoScientific™ Multiskan™ GO Microplate Spectrophotometer (Waltham, MA, USA) at a wavelength of 278 nm in case of chloramphenicol and 319 nm in case of metronidazole based on the calibration curve [38].

#### 2.2.4. Characterization

##### Thermogravimetric (TG) and Heatflow (DSC) Analysis

The thermogravimetric (TG) and heatflow (DSC) analysis of the samples were carried out with a Mettler–Toledo TGA/DSC1 instrument (Mettler–Toledo GmbH, Urdorf, Switzerland). The solid-state samples were placed in a closed aluminum crucible at a volume of 40 µL. The temperature range was 25–500 °C, and the heating rate was 10 °C/min. A nitrogen atmosphere was used (cell gas: 50 mL/min, method gas: 70 mL/min). Evaluation of the TG/DSC curves was performed with STAR^e^ software (Mettler–Toledo GmbH, Budapest, Hungary) [39].

##### Contact Angle

Contact angle measurements with the sessile drop method were performed on a DSA 30 Drop Shape Analyzer (Krüss GmbH, Hamburg, Germany) at room temperature. Drops of deionized water (5 µL) were deposited on the upper surface of the 3D printed vaginal ring (before and after the dissolution test) by an automatic dosing system. The diameter of the used needle was 0.5 mm. The contact angles were automatically calculated by Young–Laplace equation fitting on the imaged drop shape. The average contact angles were calculated from 16 drops measurements (*n* = 16) [21]. 

##### Microcomputed Tomography (MicroCT)

A SkyScan 1272 (Bruker, Kontich, Belgium) compact desktop microcomputed tomography (microCT) system was used for the measurement. Scanning parameters were as follows: image pixel size, 5 μm; matrix size, 2688 × 4032 (rows × columns); source voltage = 50 kV; source current = 200 μA; rotation step (deg) = 0.200. Flat field correction and geometrical correction were used. After scanning, SkyScan NRecon software (version 2.0.4.2) was used to reconstruct cross-section images from tomography projection images. Post-alignment, beam hardening correction, ring artifact correction, and smoothing were conducted. The output formats were DICOM and BPM images. For 3D image visualization, CTwox software (Bruker, Kontich, Belgium) was used [40].

#### 2.2.5. In Vitro Dissolution Test

The dissolution test was carried out using a modified USP Type II Erweka DT 800 dissolution apparatus (Langen, Germany) with an automatic sampling system, Ismatec IPC High-Precision Multichannel Dispenser (Wertheim, Germany). As a dissolution medium, 200 mL of simulated vaginal fluid (pH 4.2) was used based on the recipe of Owen and Katz [41]. The rotation speed was set to 60 rpm and the temperature to 37 °C. All samples were fixed to the bottom by the top part of the basket used in the USP Type I apparatus. This top part was found heavy enough to attach the sample to the bottom, but as it was composed of two parts, the samples were not affected. Samples of 2 mL were collected at 0.083, 0.25, 0.5, 1, 2, 4, 6, 8, 16, 24, 24.025, 24.5, 25, 26, 28, 30, 32, 40, 48 h using the autosampler [32,42]. 10 µL of all samples at all measurement points were put in 96-well UV-Star^®^ microplates (Greiner Bio-One Kft., Mosonmagyaróvár, Hungary) and were diluted with 190 µL of SVF. The absorbance of the release drug was determined by UV spectrophotometry with Thermo Scientific™ Multiskan™ GO Microplate Spectrophotometer at a wavelength of 278 nm in case of chloramphenicol and 319 nm in case of metronidazole. Dissolution experiments were conducted with four replicates [43,44].

To compare the dissolution data of the different samples, similarity and difference factors were calculated; as a model-independent approach, the difference—*f*1 factor and similarity—*f*2 factor was calculated for each sample:

$$f1=\frac{\sum_{j=1}^{n}\left(Rj-Tj\right)}{\sum_{j=1}^{n}Rj}\times 100,$$

where *n* is the number of samples and *Rj* and *Tj* are the percent dissolved of the reference and the test products at time point *j*.

$$f2=50\times \log\{{\left(1+(\frac{1}{n})\sum_{j=1}^{n}wj\;{\left(Rj-Tj\right)}^{2}\right)}^{-0.5}\times 100\},$$

where *wj* is an optional weight factor.

For the determination of release kinetics, the dissolved API amount was fitted to zero-order and first-order model equations:

$$Q=Q_{0}+k_{0}t$$

$$Q_{t}=Q_{0}\times e^{-k_{1}t},$$

where *Q* is the amount of drug released at time *t*, *Q*_0_ is the initial amount of the drug, and *Q_t_* is the amount of drug remaining at time *t*. *k*_0_ and *k*_1_ are the kinetic constants for the zero-order and first-order models, respectively [40].

#### 2.2.6. Biocompatibility Experiments

##### Cytotoxicity Experiments

1. Sterilization

The 3D-printed carrier systems were immersed in 70% (*v/v*) ethanol in a laminar air flow (LAF) cabinet for 12 h, and were individually placed to sterile medical-grade paper for drying to avoid the infection [43].

2. Cell Culture

The human Negroid cervix epithelioid carcinoma (HeLa) cell line was received from the European Collection of Cell Cultures (ECACC, Salisbury, UK, catalog no. 93031013), which is a well-established cell culture from ECACC, and the protocol is based on the authors’ research. Cells were seeded in plastic cell culture flasks (Thermo Fisher Scientific Inc., Budapest, Hungary) in DMEM medium supplemented with 3.7 g/L NaHCO3, 10% (*v/v*) heat-inactivated fetal bovine serum (FBS), 1% (*v/v*) nonessential amino acid solution, 1% (*v/v*) l-glutamine, 100 IU/mL penicillin and 100 µg/mL streptomycin at 37 °C in an atmosphere of 5% CO_2_. For the cytotoxicity experiments, cells with 20 to 40 passages were used. The cells were passaged every three or four days [45].

3. MTT Cell Viability Assay

Cells were seeded on flat-bottomed 96-well tissue culture plates at a density of 10^4^ or 3 × 10^4^ cells/well. After separate sterilization of the test samples, they were put in sterile centrifuge tubes, immersed in 20 mL of DMEM medium, and stored in a cell incubator at 37 °C. The tests were performed on 4th, 8th, and 12th days, and the samples were stored under the same conditions. The first step of the MTT assay was to remove the culture media from the cells, and then the cells were treated with 200 µL of the test sample solution and incubated for 30 min. After the incubation, the samples were removed, and the cells were washed with 200 µL PBS solution/well. Then, the cells were incubated with 100 µL 0.5 mg/mL MTT dye (3-(4,5-dimethylthiazol-2-yl)-2,5-diphenyl-2H-tetrazolium bromide dye) for at least 3 h. Finally, the formazan crystals were dissolved in acidic isopropanol (isopropanol: 1.0 N hydrochloric acid = 25:1). The absorbance was measured at 570 nm against a 690 nm reference with a FLUOstar OPTIMA Microplate Reader (BMG LABTECH, Offenburg, Germany). Cell viability was expressed as the percentage of the untreated control [46].

4. Microbiological Evaluation

Candida albicans SC5314 reference strain and Escherichia coli ATCC 25922 strain were used in this study. Colonies were subcultured on Sabouraud dextrose agar and Mueller-Hinton agar for *C. albicans* and *E. coli*, respectively. The growth of the individual strains in coculture was evaluated to examine the effect of vaginal rings containing different compounds. The tested samples were as follows: (a) empty vaginal ring; (b) metronidazole containing vaginal ring; (c) 3 *w/w*% chitosan and 4 *w/w*% hydroxyethyl cellulose-containing vaginal ring; (d) 3 *w/w*% chitosan and metronidazole containing vaginal ring.

The final cell concentrations were adjusted to 2–5 × 10^5^ cells/mL in RPMI-1640 broth + Mueller-Hinton broth (50:50 *v/v*%) both for *C. albicans* and *E. coli*, respectively. The total broth volume was 20 mL in each flask. Flasks were incubated with agitation in darkness at 35 °C. A total of 100 μL were removed at 0, 2, 4, 6, 8, 10, 12, and 24 h, serially diluted 10-fold in sterile physiological saline then plated onto either Sabouraud dextrose agar supplemented with 8 mg/L metronidazole and Mueller-Hinton agar supplemented with 8 mg/L amphotericin B. Plates were incubated for 24 and 48 h at 35 °C for *E. coli* and *C. albicans*, respectively.

#### 2.2.7. Statistical Analysis

Data were analyzed using GraphPad Prism (version 7.0; GraphPad Software, Inc., San Diego, CA, USA) and presented as means ± standard deviation (SD). If it is not mentioned in the method description, then the experiments were carried out in triplicate [46].

## 3. Results

### 3.1. Design of the Drug Reservoirs and Printing of the Samples

**Figure 1:**
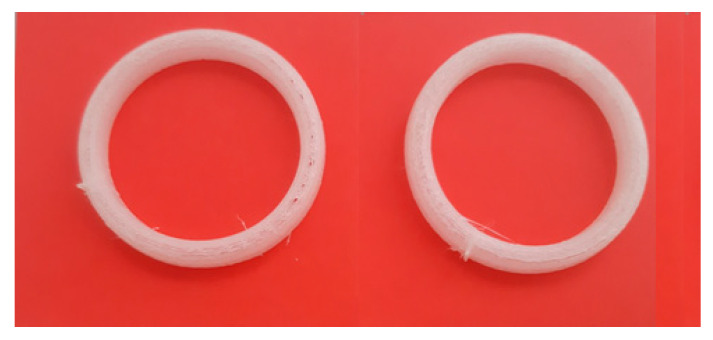
Image of the printed samples.

In our experiments, four different polymers, polylactic acid (PLA), PLA Gypsum PLA Foam, and thermoplastic polyurethane (TPU) were used for the 3D printing. The sample thickness was 5 mm, and the diameter was 39.39 and 41.39 (Figure 1). At the beginning of our research, we started our experiments on different PLA filaments but based on our performed experiments, none of them proved to be flexible enough for the requirement of a vaginal ring. Based on our results, TPU showed adequate texture properties, which were confirmed by the manufacturer as well. Then the printing parameters were determined. In the case of the TPU the 32nd printing parameter adjustment was acceptable for the manufacturing.

### 3.2. Gel Formation and Sample Manufacturing

The 1st formulation with Carbopol 934 was found inappropriate for our experiments because the gel was too watery in the applied *w/w*% and was not soluble in the simulated vaginal fluid. The 2nd formulation with 3 *w/w*% chitosan was consistent and homogenous. The 3rd formulation was more watery than 2nd formulation, but both formulations could be dissolved in SVF. The 4th formulation was a semisolid gel after cooling.

**Figure 2:**
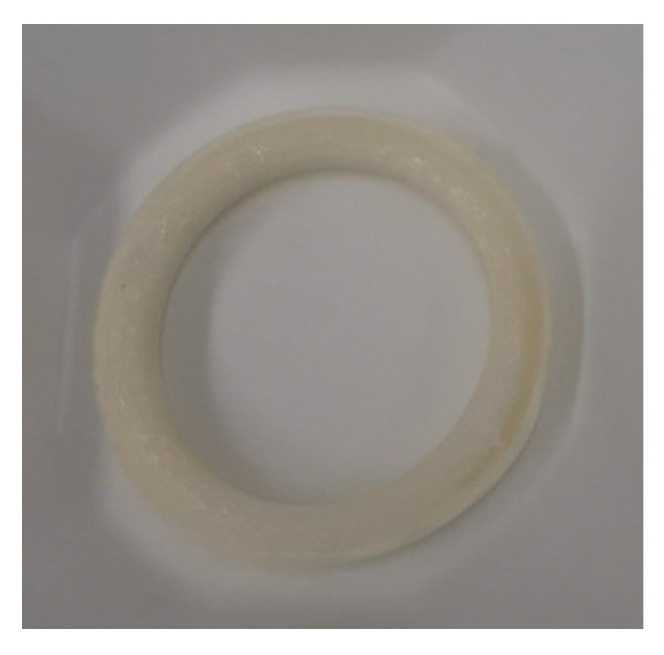
Image of the manufactured samples.

The API’s were suspended with the gels and manually filled into the pre-printed vaginal rings. The samples fitting was adequate (Figure 2).

### 3.3. Weight Variation and Content Uniformity

The measured weight and the content uniformity results can be seen in Table 4. The samples’ average weight (g) was similar in all cases. The samples were filled with 1.5 g API containing jellified agent.

### 3.4. Characterization

#### 3.4.1. Thermogravimetric (TG) and Heatflow (DSC) Analysis

**Figure 3:**
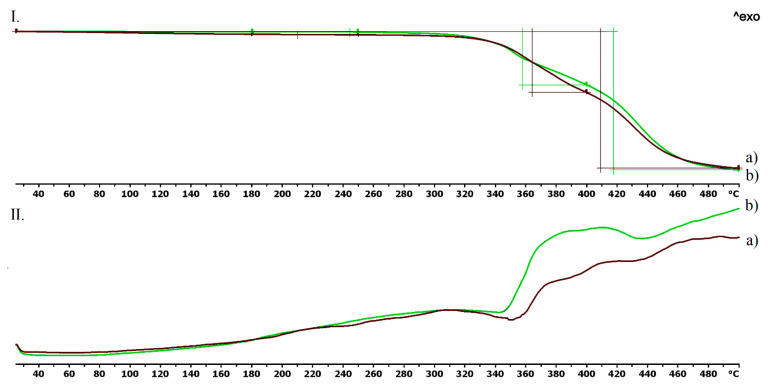
Thermogravimetric (**I**) and heatflow (**II**) analysis of the TPU filament (**a**) and the empty vaginal ring (**b**). Thermal behavior was analyzed between 25 °C and 500 °C.

The TG curve of the TPU filament (Figure 3(I.a)) and empty vaginal ring (Figure 3(I.b)) showed thermal stability until 300–320 °C, and no kind of difference could be seen until 370 °C, thus, the used polymer is stable at the applied printing temperature. The decomposition was around 39% until 400 °C and 89% until 500 °C. In the case of the DSC curves, neither a characteristic endothermic peak nor an exothermic peak could be determined. In the case of the TPU filament, there was a flattened endothermic peak at 352 °C when the decomposition started (Figure 3(II.a)). In the case of the empty vaginal ring, the decomposition started after 348 °C, and a flattened exothermic peak could be determined (Figure 3(II.b)).

**Figure 4:**
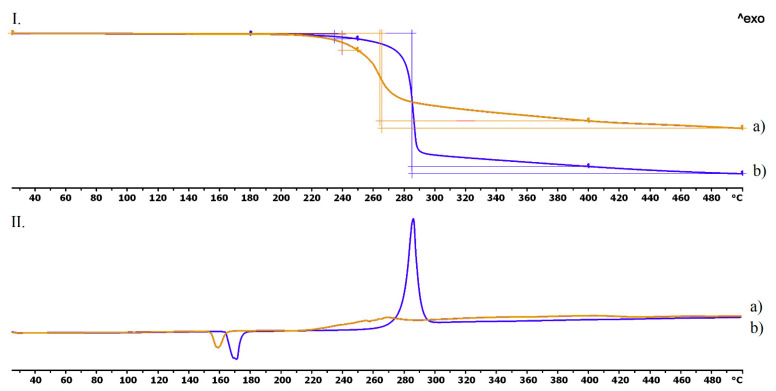
Thermogravimetric (**I**) and heatflow (**II**) analysis of the chloramphenicol (**a**) and the metronidazole (**b**). Thermal behavior was analyzed between 25 °C and 500 °C.

The thermogravimetric and heatflow analysis of the chloramphenicol and metronidazole could be seen in Figure 4. The curves proved that chloramphenicol was stable until 210 °C and metronidazole until 220 °C. In the case of chloramphenicol the decomposition was around 56% until 500 °C, and metronidazole’s was around 84% until 500 °C (Figure 4I). The DSC curve showed an endothermic peak at 159 °C in the case of the chloramphenicol, which is the melting point and not followed by a mass decrease until 210 °C (Figure 4(II.a)). On the DSC curve of the metronidazole, an endothermic peak at 170 °C could be characterized without a mass decrease. A characteristic exothermic peak was determined at 288 °C, which was followed by decomposition (Figure 4(II.b)).

**Figure 5:**
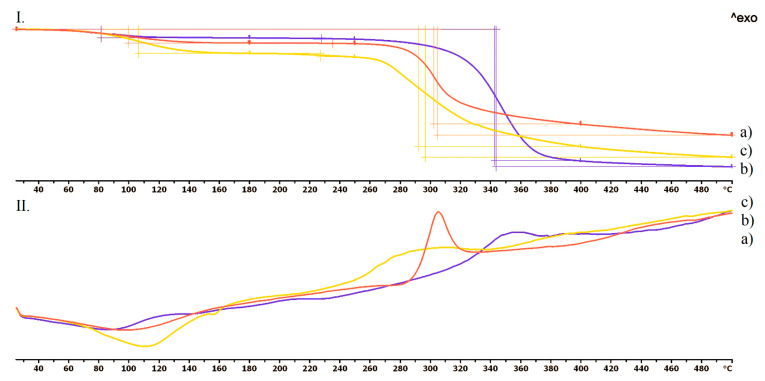
Thermogravimetric (**I**) and heatflow (**II**) analysis of the chitosan (**a**), hydroxyethyl cellulose (**b**) and agar-agar (**c**). Thermal behavior was analyzed between 25 °C and 500 °C.

In Figure 5, the three jellifying agents: chitosan (a), hydroxyethyl cellulose (b), and agar-agar (c) thermogravimetric and heatflow analysis could be seen. In the case of the chitosan, an extended endothermic peak was realized, and an 8% decomposition was detected until 180 °C. Based on our experiments, this was due to the evaporation of the surface absorbed moisture. The decomposition took place in one step right after the exothermic peak at 306 °C (Figure 5(II.a)). Hydroxyethyl cellulose showed a 5% decomposition until 180 °C, which was the evaporation of the absorbed water as well. The flattened exothermic peak was around 351 °C. The decomposition was around 79% until 500 °C (Figure 5(II.b)). Agar-agar was stable until 260 °C, when a flattened exothermic peak started. The decomposition was around 74% until 500 °C. Agar-agar showed a 13% decomposition until 180 °C, which was the consequence of the evaporation.

#### 3.4.2. Contact Angle

**Figure 6:**
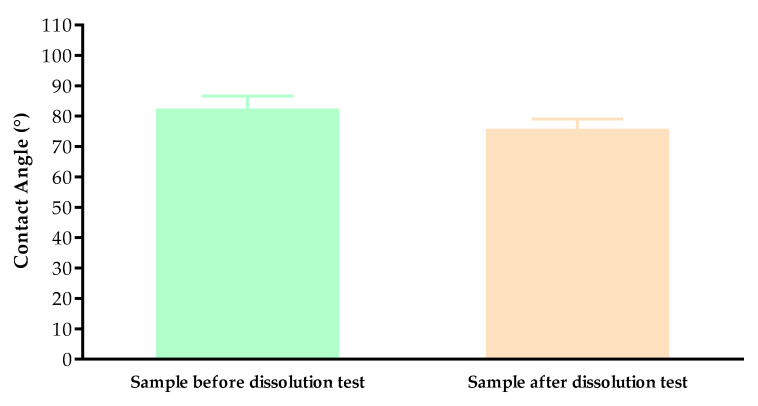
Contact angle values of the printed TPU samples before the dissolution test and the filled samples after the dissolution test. Data are expressed as means ± SD. Experiments were performed sixteen times, *n* = 16.

The contact angle values are given in Figure 6. The contact angle values were measured on the surface of the printed vaginal ring samples before and after the dissolution test. The two different samples had different contact angle values: before the dissolution test 82.48 and after the dissolution test 75.84. The results were statistically analyzed, and a significant difference **** was found (*p* < 0.0001).

#### 3.4.3. Microcomputed Tomography (MicroCT)

**Figure 7:**
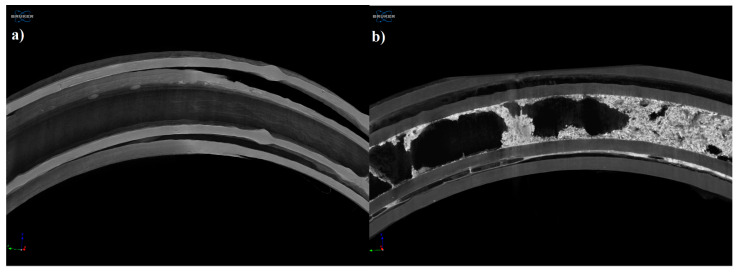
Reconstructed microCT image from a vertical cut of the printed sample (**a**) and examined sample after the dissolution test (**b**). Image pixel size is 5 μm.

The samples were examined by microcomputed tomography (microCT) before and after the dissolution test to determine their morphology. Figure 7 represents the printed sample (a) and the examined sample after the dissolution test (b). The localization of the remaining gel could be clearly seen after the dissolution test.

**Figure 8:**
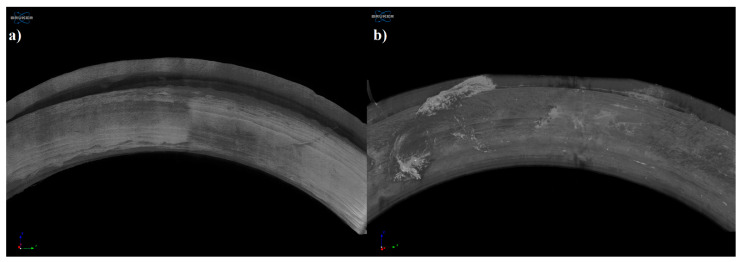
Reconstructed microCT image from the upper surface of the printed sample (**a**) and examined sample after the dissolution test (**b**). Image pixel size is 5 μm.

Figure 8 represents the upper surface of the printed sample (a) and examined sample after the dissolution test (b). On the surface of the dissolved sample, no kind of change or alteration was detected.

### 3.5. In Vitro Dissolution Test

The dissolution profiles of the samples were determined by a modified USP type II in vitro dissolution apparatus to determine the dissolution profiles. The dissolved API amount (%) at every sampling time and the standard deviation (±SD) results can be found in Appendix A, but the dissolved API amount (%) at 2 h, 8 h, and 48 h can be found in Table 5. At 2 h, the dissolved API amount varied from 0% to 39.89% and at 8 h from 0% to 52.31%, respectively. The dissolution from the chloramphenicol 4th formulation did not start in the first 8 h. At 48 h, we found that the dissolved API amount varied between 2.14% and 47.98% depending on the API and the jellifying agent type.

**Figure 9:**
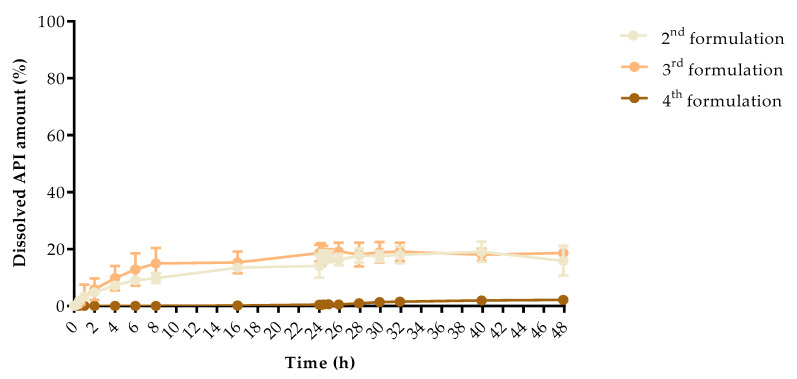
Dissolved API amount (%) versus time (h) for samples jellified with chloramphenicol; mean ± SD, *n* = 4.

**Figure 10:**
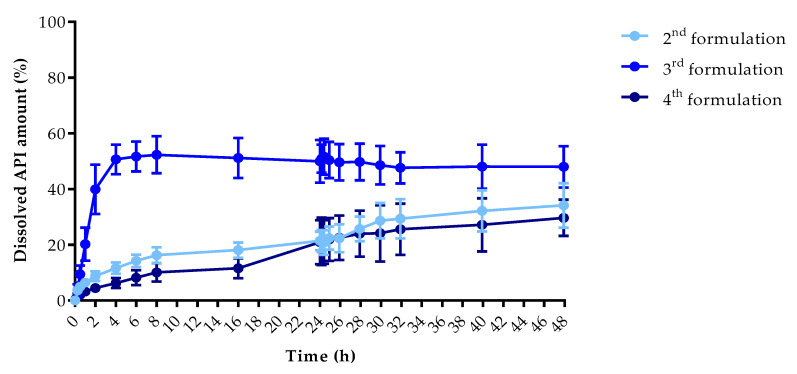
Dissolved API amount (%) versus time (h) for samples jellified with metronidazole; mean ± SD, *n* = 4.

The dissolved API amount (%) was plotted against the time (h) for the chloramphenicol (Figure 9) and metronidazole samples (Figure 10). The results are means ± SD, *n* = 4.

Pairwise comparison results of the different samples dissolution profiles can be found in Appendix B: *f*1 value of the difference factor calculation and *f*2 value of the similarity value calculation. The interpretation was based on the FDA guideline. We found that all of our sample dissolution profile was considered non-similar. Based on this comparison, all samples had different dissolution curves.

Drug release data were fitted to zero-order and first-order models (Table 6). Determination coefficients were used to determine the best fit. The metronidazole 2nd formulation sample fitted to the first-order model, which confirmed the linear curve shape (Figure 10). The calculations revealed that other samples could not be fitted to these models if we compared the results from 0–48 h. In the case of metronidazole 4th formulation, the results were the same, thus, we decided not to interpret a conclusion from it. The dissolved API amounts were fitted to the same kinetic models, but separately between 0–8 h and 8–24 h. From the chloramphenicol 4th formulation, the API was not dissolved in the first 8 h, thus, the kinetic determination is inadequate, but all other samples fitted to the first-order kinetic model. In the 8–48 h interval the chloramphenicol 4th formulation and metronidazole 2nd formulation can be fitted to first-order kinetic model.

### 3.6. Biocompatibility Experiments

#### 3.6.1. Cytotoxicity Experiments

**Figure 11:**
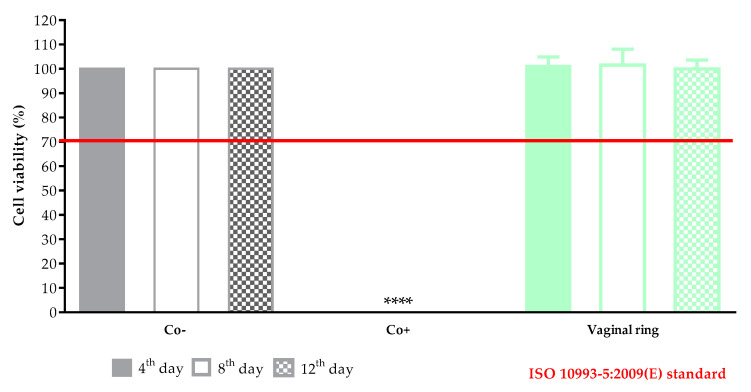
The prolonged cytotoxicity of the 3D printed sample byTPU. MTT cell viability tests were performed on days 4, 8, and 12. Cell viability was expressed as the percentage of untreated control (Co−). As a positive control (Co+), Triton X 100 (10% *w*/*v*) was used. Data were expressed as means of four independent experiments ± SD.

A prolonged cell viability test was performed to gain information about the cytocompatibility of the 3D printed vaginal rings. The samples were incubated in the cell culture medium for 4, 8, and 12 days, and the monolayer formed by HeLa cells was treated with this medium to determine if any kind of xenobiotic was dissolved from the sample. This method differed from the original MTT assay because the cell viability was expressed as the percentage of negative or untreated control (DMME medium, Co-) in harmonization with the ISO standard. As a positive control (Co+), Triton-X 100 (10% *w/v*) solubilizing agent was used, which had significant differences from the two other examined samples. Based on the ISO 10993-5:2009(E) standard, if the relative cell viability was higher than 70% in comparison with the control group (100%), the materials could be considered non-cytotoxic [47]. According to this regulation, the printed vaginal ring sample could be qualified as cytocompatible (Figure 11).

#### 3.6.2. Microbiological Evaluation

**Figure 12:**
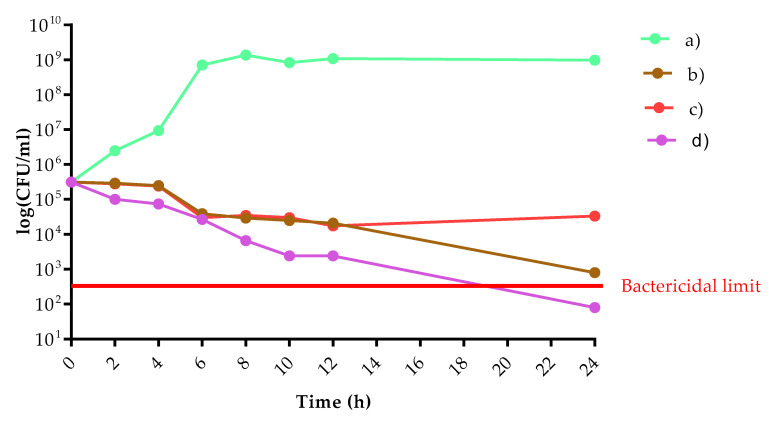
The logarithmical colony-forming unit (CFU)/mL in case of *E. coli* versus time (h) where (**a**) empty vaginal ring; (**b**) metronidazole containing vaginal ring; (**c**) 3 *w/w*% chitosan and 4 *w/w*% hydroxyethyl cellulose-containing vaginal ring; (**d**) 3 *w/w*% chitosan and metronidazole containing vaginal ring.

**Figure 13:**
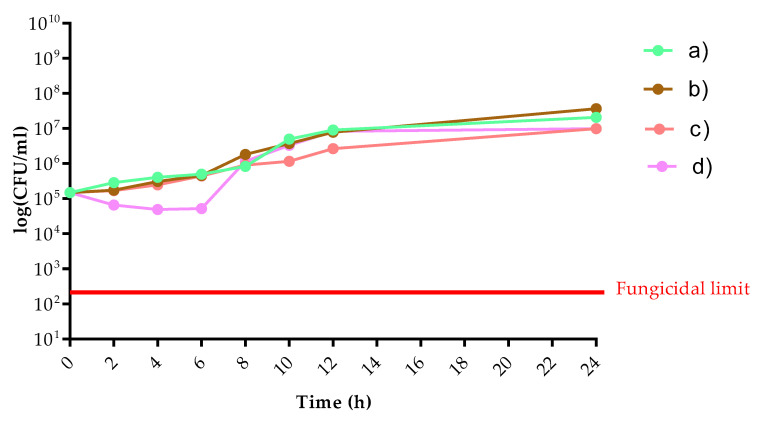
The logarithmical colony-forming unit (CFU)/mL in case of *C. albicans* versus time (h) where (**a**) empty vaginal ring; (**b**) metronidazole containing vaginal ring; (**c**) 3 *w/w*% chitosan and 4 *w/w*% hydroxyethyl cellulose containing vaginal ring; (**d**) 3 *w/w*% chitosan and metronidazole containing vaginal ring.

*E. coli* cell count showed a significant decrease in the presence of three tested rings. It is noteworthy that the ring containing chitosan with metronidazole in combinations produced a bactericidal effect at 24 h. While the metronidazole alone produced only a static effect after 24 h (Figure 12). In case of *C. albicans* the ring containing chitosan with metronidazole exerted a weak fungistatic effect between 2 and 6 h. However, the observed cell counts between 8 and 24 h were similar compared to untreated control cells (Figure 13).

## 4. Discussion

In our study, vaginal rings were manufactured with 3D printing from commercially available polymer filaments. Then, the vaginal ring samples as carrier systems were filled with jellified chloramphenicol or metronidazole. The reason for these investigations were to certify the applicability of FDM printing in the field of personalized medication manufacturing. The APIs can be delivered at the right dose locally in the vagina, which provide an excellent response [48,49].

3D printing significantly speeds up the design cycle, both in development and in industrial manufacturing [29]. One of the newest articles published in 2021 was about the manufacturing of a 3D printed vaginal ring from thermoplastic polyurethane filled with clotrimazole to treat vulvovaginal candidiasis. Even though the two research groups had the same ideas but with different manufacturing processes and API, Tiboni et al. used hot-melt extrusion for the manufacturing of the clotrimazole containing filament [50]. Based on another research, the endothermic peak of the clotrimazole on the DSC curve was at 145.6 °C but without decomposition until 160 °C [51].

In our previous article, we already bothered with the problem of the required high printing temperature and how difficult it can be to fill the samples with the API [29]. One study showed that the APIs’ weight loss was around 5% after the FDM printing, but in the pharmaceutical industry, only a maximum of 1% API deviation could be accepted [52]. Our research group developed an easy method to fill the samples with the API. Furthermore, the amount and stability of the API were not affected. As it is a pre-printed carrier system, API can be directly applied in the desired amount to the immediately printed drug delivery system, and the patient can start the application earlier with the hope of a better outcome.

The thermogravimetric and heatflow analysis of the TPU filament and empty vaginal ring showed thermal stability until 300–320 °C, thus the used polymer is stable at the applied printing temperature. The TGA curves proved that chloramphenicol was stable until 210 °C and metronidazole until 220 °C. The three jellifying agents: chitosan, hydroxyethyl cellulose, and agar-agar were stable on the printing temperatures. These results are in agreement with the scientific data [53,54]. Based on these results, these API’s should not be mixed with the TPU polymer because the applied printing temperature was 233 °C. Our method was adequate because the API’s were added after the vaginal ring was pre-printed.

Contact angle results showed that the printed sample and the sample after dissolution test had different wettability properties and after the dissolution test in simulated vaginal fluid, the sample was more hydrophilic than before the test. Based on the results, our samples can be identified as moderate with wettability properties, which can affect the samples’ mucoadhesion properties in the vagina [55].

Based on the dissolution profiles, the dissolved API amount varied from 0% to 52.31% at 8 h. The dissolution from the chloramphenicol 4th formulation did not start in the first 8 h. The dissolved API amount was higher from the metronidazole-containing samples than the chloramphenicol-containing samples. If we compare the jellifying agents, the 3 *w/w*% chitosan showed the highest dissolution amount and agar-agar the less within 48 h.

Based on the microCT results, the jellified API was just partly dissolved from the vaginal ring, which is in correlation with our dissolution test results. On the surface of the dissolved sample, no kind of change or alteration was detected.

The dissolution from drug delivery systems depends on various physical and chemical properties, which results in difficulties at describing proper mathematical models [56], thus, our samples were examined with first-order and zero-order kinetics at different time intervals [57,58]. All of our samples could be fitted to first-order kinetics model independently from the used API or auxiliary material [59].

The in vitro cytotoxicity profile of the samples was determined with a long-term MTT assay. The method was harmonized with the ISO 10993 standard but with a shorter incubation period [60]. The sterile samples were stored in DMEM medium at 37 °C and the potentially dissolved xenobiotics were measured by MTT assay on days 4, 8, and 12. The in vitro cytotoxicity method as a compulsory test can be the first filter through the determination of biocompatibility and can be a good predictor of the in vivo results [29,61]. Based on the prolonged MTT assay on the HeLa cell line, results were in accordance with the ISO 10993:5 standard, and the printed vaginal ring samples can be considered cytocompatible.

The microbiological evaluation is part of the biocompatibility determination, and with the results of the MTT assay can give us a good prediction about the in vivo data. We determined the effect against *E. coli* and *C. albicans* as reference isolates [62]. *E. coli* cell count showed a significant decrease in the presence of all three tested rings. The vaginal ring that contains chitosan with chloramphenicol in combinations produced a bactericidal effect at 24 h. While the chloramphenicol alone produced only a static effect after 24 h. Thus, we can state that the previously well-described effect of chitosan can help to suppress bacterial vaginosis [63,64].

In conclusion, vaginal rings from thermoplastic polyurethane were successfully 3D printed by FDM technology. The pre-printed samples were filled with chloramphenicol or metronidazole and jellified with chitosan/HEC or agar-agar. The special experimental arrangement ensures that all kinds of API can be utilized during printing without heat damage or API loss, confirmed by TGA results. Based on the dissolution curves, the used API and jellifying agent can modify the dissolved API amount. Based on the MTT assay results, TPU polymer can be considered cytocompatible. The microbiological evaluation confirmed that metronidazole and chitosan have a synergistic effect against *E. coli*. Based on the overall project, TPU polymer filled with metronidazole was suggested for further investigations.

## Figures and Tables

**Table 1:** Properties of the commercially available filaments.

Properties	Method	PLA	PLA Gypsum	PLA Foam	TPU
Specific gravity (g/cm^3^)	D792	1.24	1.25	1.00	1.2
Heat distortion temperature at 0.45 MPa (°C)	D790	55	55	55	106
Glass Trans. temperature (°C)	D3418	55–60	55–60	55–60	150–230
Tensile strength (MPa)	ISO 527	60	54	53	No break
Tensile modulus (MPa)	ISO 527	3800	3200	6040	No break
Notched Izod impact (kJ/m^2^)	ISO 180	16	14	12	No break

**Table 2:** Properties of the 3D-printed filaments.

Properties	Method	PLA	PLA Gypsum	PLA Foam	TPU
Tensile strength (MPa)	ISO 527	31.6	25.0	20.0	No break
Tensile modulus (GPa)	ISO 527	1.8	1.4	5.1	No break
Notched Izod impact (kJ/m^2^)	ISO 180	2.6	2.9	2.2	No break

**Table 3:** Printing characteristics of the samples.

Filament Type	PLA	PLA Gypsum	PLA Foam	TPU
Filament Diameter (mm)	1.75	1.75	1.75	1.75
Extruder Nozzle Diameter (µm)	400	400	400	400
Infill Percentage (%)	0	0	0	0
Extrusion Temperature (°C)	215	215	215	233
Bed Temperature (°C)	60	60	60	65
Layer Thickness (µm)	100	100	100	100

**Table 4:** The samples’ average weight (g) with standard deviation (SD) and the average content uniformity (g) results with SD in case of the three different formulations containing chloramphenicol and metronidazole.

Sample		Weight		Content Uniformity	
		Average (g)	±SD	Average (g)	±SD
2nd formulation	Chloramphenicol	2.83	0.42	1.51	0.20
3rd formulation		2.82	0.38	1.49	0.14
4th formulation		2.86	0.24	1.52	0.23
2nd formulation	Metronidazole	2.81	0.41	1.52	0.01
3rd formulation		2.84	0.12	1.48	0.25
4th formulation		2.84	0.36	1.50	0.11

**Table 5:** The dissolved API amount (%) results at 2 h, 8 h, and 48 h can be found in the table and the other sampling time results in Appendix A, with the standard deviation results.

Sample		2 h		8 h		48 h	
		Dissolved API Amount (%)	±SD	Dissolved API Amount (%)	±SD	Dissolved API Amount (%)	±SD
Chloramphenicol	2nd formulation	4.79	1.36	9.76	1.70	15.86	5.10
	3rd formulation	5.89	3.77	14.93	5.42	18.57	2.51
	4th formulation	0.00	0.00	0.00	0.00	2.14	0.92
Metronidazole	2nd formulation	8.76	1.74	16.31	2.79	34.12	7.97
	3rd formulation	39.89	8.87	52.31	6.64	47.98	7.42
	4th formulation	4.47	0.72	10.11	3.33	29.67	6.50

**Table 6:** The kinetic analysis of the dissolved samples. Drug release data were fitted to zero-order and first-order kinetic models for 0–48 h, 0–8 h, and 8–48 h.

Sample		Zero-Order Kinetics	First-Order Kinetics	Zero-Order Kinetics	First-Order Kinetics	Zero-Order Kinetics	First-Order Kinetics
		0–48 h	0–48 h	0–8 h	0–8 h	8–48 h	8–48 h
Chloramphenicol	2nd formulation	0.808	0.806	0.922	0.925	0.177	0.176
	3rd formulation	0.730	0.726	0.967	0.971	0.215	0.214
	4th formulation	0.765	0.763	0.000	0.000	0.870	0.871
Metronidazole	2nd formulation	0.921	0.944	0.891	0.978	0.902	0.910
	3rd formulation	0.410	0.361	0.796	0.818	0.635	0.632
	4th formulation	0.896	0.896	0.950	0.973	0.465	0.473

## Appendix A

**Sample**	**Chloramphenicol**						**Metronidazole**					
	**2nd Formulation**		**3rd Formulation**		**4th Formulation**		**2nd Formulation**		**3rd Formulation**		**4th Formulation**	
**Sampling Time (h)**	**Dissolved API (%)**	**±SD**	**Dissolved API (%)**	**±SD**	**Dissolved API (%)**	**±SD**	**Dissolved API (%)**	**±SD**	**Dissolved API (%)**	**±SD**	**Dissolved API (%)**	**±SD**
0	0.00	0.00	0.00	0.00	0.00	0.00	0.00	0.00	0.00	0.00	0.00	0.00
0.083	0.00	0.00	0.00	0.00	0.00	0.00	0.00	0.17	0.05	0.09	0.67	0.66
0.25	0.36	0.49	0.50	0.74	0.00	0.00	3.63	0.75	3.70	2.16	2.57	1.07
0.5	1.77	1.01	0.76	0.84	0.00	0.00	4.98	1.10	9.48	3.07	1.99	0.33
1	3.36	1.01	3.31	4.15	0.00	0.00	6.42	1.00	20.20	5.90	3.18	0.47
2	4.79	1.36	5.89	3.77	0.00	0.00	8.76	1.74	39.89	8.87	4.47	0.72
4	7.21	1.23	9.75	4.33	0.00	0.00	11.63	2.02	50.64	5.32	6.25	1.82
6	9.01	0.75	12.81	5.71	0.00	0.00	14.24	2.24	51.68	5.37	8.18	2.65
8	9.76	1.70	14.93	5.42	0.00	0.00	16.31	2.79	52.31	6.64	10.11	3.33
16	13.44	0.92	15.32	3.83	0.16	0.32	18.08	2.69	51.15	7.16	11.54	3.55
24	14.11	4.15	18.60	2.85	0.43	0.58	21.40	3.35	49.99	7.66	20.92	7.86
24.083	17.45	1.54	18.10	4.02	0.35	0.41	21.33	3.93	50.91	5.04	21.19	8.52
24.25	17.15	2.44	18.10	3.13	0.53	0.52	20.79	4.44	51.63	6.43	20.98	7.80
24.5	17.36	2.02	18.22	1.77	0.61	0.54	22.42	4.15	50.42	6.49	21.90	7.66
26	16.31	2.03	19.17	3.06	0.53	0.57	22.34	5.01	49.63	6.55	22.51	7.98
28	17.69	2.42	18.08	4.19	0.98	0.79	25.71	4.43	49.73	6.56	23.98	8.26
30	17.49	1.86	18.91	3.59	1.40	0.95	28.65	6.32	48.55	6.87	24.13	10.07
32	17.94	2.96	19.06	3.20	1.52	0.68	29.34	7.08	47.61	5.57	25.57	9.22
40	19.03	3.60	18.04	2.14	1.96	0.64	32.15	7.38	48.05	7.92	27.16	9.51
48	15.86	5.10	18.57	2.51	2.14	0.92	34.12	7.97	47.98	7.42	29.67	6.50

## Appendix B

**Pairwise Comparison of Dissolution Profiles**			
**Sample 1 vs.**	**Sample 2**	***f*1**	***f*2 (%)**
Chloramphenicol 2nd formulation	Chloramphenicol 3rd formulation	13.32	76.07
Chloramphenicol 2nd formulation	Chloramphenicol 4th formulation	2156.98	39.86
Chloramphenicol 2nd formulation	Metronidazole 2nd formulation	35.74	50.57
Chloramphenicol 2nd formulation	Metronidazole 3rd formulation	71.56	19.96
Chloramphenicol 2nd formulation	Metronidazole 4th formulation	24.55	61.43
Chloramphenicol 3rd formulation	Chloramphenicol 4th formulation	2434.11	37.36
Chloramphenicol 3rd formulation	Metronidazole 2nd formulation	27.84	53.95
Chloramphenicol 3rd formulation	Metronidazole 3rd formulation	68.07	21.20
Chloramphenicol 3rd formulation	Metronidazole 4th formulation	24.88	63.33
Chloramphenicol 4th formulation	Metronidazole 2nd formulation	97.15	30.20
Chloramphenicol 4th formulation	Metronidazole 3rd formulation	98.74	13.00
Chloramphenicol 4th formulation	Metronidazole 4th formulation	96.53	33.80
Metronidazole 2nd formulation	Metronidazole 3rd formulation	55.75	24.62
Metronidazole 2nd formulation	Metronidazole 4th formulation	22.79	62.45
Metronidazole 3rd formulation	Metronidazole 4th formulation	176.28	21.99
